# LncRNA PVT1 promotes cervical cancer progression by sponging miR-503 to upregulate ARL2 expression

**DOI:** 10.1515/biol-2021-0002

**Published:** 2021-01-21

**Authors:** Weiwei Liu, Dongmei Yao, Bo Huang

**Affiliations:** Department of Gynecology, Maternal and Child Health Hospital of Hubei Province, Wuhan, Hubei, 430070, China; Department of Gynaecology and Obstetrics, Hubei General Hospital, No. 99 ZhangZhiDong Street, Wuchang District, Wuhan, Hubei, 430060, China

**Keywords:** lncRNA PVT1, miR-503, ARL2, cervical cancer, progression

## Abstract

Cervical cancer (CC) is a huge threat to the health of women worldwide. Long non-coding RNA plasmacytoma variant translocation 1 gene (PVT1) was proved to be associated with the development of diverse human cancers, including CC. Nevertheless, the exact mechanism of PVT1 in CC progression remains unclear. Levels of PVT1, microRNA-503 (miR-503), and ADP ribosylation factor-like protein 2 (ARL2) were measured by quantitative reverse transcription-polymerase chain reaction or western blot assay. 3-(4,5)-Dimethylthiazole-2-y1)-2,5-biphenyl tetrazolium bromide (MTT) and flow cytometry were used to examine cell viability and apoptosis, respectively. For migration and invasion detection, transwell assay was performed. The interaction between miR-503 and PVT1 or ARL2 was shown by dual luciferase reporter assay. A nude mouse model was constructed to clarify the role of PVT1 *in vivo*. PVT1 and ARL2 expressions were increased, whereas miR-503 expression was decreased in CC tissues and cells. PVT1 was a sponge of miR-503, and miR-503 targeted ARL2. PVT1 knockdown suppressed proliferation, migration, and invasion of CC cells, which could be largely reverted by miR-503 inhibitor. In addition, upregulated ARL2 could attenuate si-PVT1-mediated anti-proliferation and anti-metastasis effects on CC cells. Silenced PVT1 also inhibited CC tumor growth *in vivo*. PVT1 knockdown exerted tumor suppressor role in CC progression via the miR-503/ARL2 axis, at least in part.

## 1 Introduction

Cervical cancer (CC) is the third most common cancer and the fourth most deadly malignancy among women in the world, with approximately 5.30 × 10^5^ new CC cases and 2.75 × 10^5^ CC-induced deaths every year [1,2]. Recently, prognosis and treatment approaches of CC have developed greatly. Nevertheless, routine treatment methods, including surgery, chemotherapy, and radiotherapy, have not obviously elevated the 5-year survival rate of advanced patients, because of metastasis, recurrence, and drug resistance [3,4]. Therefore, it is urgently imperative to deeply understand the occurrence, progression, and treatment of CC so as to explore more efficient treatment approaches.

According to the studies executed by most scholars, long non-coding RNAs (lncRNAs), non-coding RNAs longer than 200 nucleotides (nts), have been confirmed as major regulators in many human diseases, such as cancers [5,6,7]. Actually, numerous lncRNAs exhibit dysregulated expression in CC and are strongly associated with tumorigenesis, progression, and prognosis of CC. For example, LINC00511 served as an oncogene in CC, and it had the potential to be an efficient biomarker and therapeutic target for patients with CC [8]. A former study indicated that lncRNA CCHE1 was considerably upregulated in CC tumor tissues, and it was identified as a prognostic biomarker and novel treatment target [9]. LncRNA maternally expressed 3 (MEG3) effectively repressed the tumor formation ability of CC cells *in vivo* and hampered proliferation, whereas it elevated apoptosis of CC cells *in vitro* [10]. LncRNA plasmacytoma variant translocation 1 gene (PVT1), located at 8q24.21, was significantly upregulated and therefore identified as an oncogene in the progression of diverse human cancers, such as non-small cell lung cancer (NSCLC) [11], pancreatic cancer [12], esophageal cancer [13], and CC [14]. However, the functional impact of PVT1 on CC progression has not been fully elucidated.

MicroRNAs (miRNAs), a group of short endogenous non-coding RNAs with 19–25 nts, can bind to the 3′-untranslated regions (3′-UTRs) to trigger target mRNA repression at the posttranscriptional level [15,16]. In the past few decades, a large number of miRNAs were manifested to participate in the regulation of the development and progression of CC [17,18]. In addition, ectopic expression of microRNA-503 (miR-503) was reported to inhibit the development and progression of certain human cancers, including CC [19]. Whether miR-503 was involved in PVT1-mediated CC development remains uncertain.

ADP-ribosylation factor-like protein 2 (ARL2), a member of the ADP-ribosylation factor (ARF) family, is a highly conserved gene located on chromosome 11 (11q13) [20,21]. ARL2 could serve as a target of miR-497-5p to affect the osteosarcoma (OS) development [22], and a downstream gene of miR-214 to mediate colon cancer progression [23]. In addition, ARL2 could perform as prognostic or therapeutic target for CC [24]. However, whether there was an interaction between PVT1 and ARL2 in CC progression is unclear.

In the current research, expression level of PVT1, its functional impact on the proliferation, apoptosis, and metastasis of CC cells *in vitro*, and on tumor growth *in vivo*, as well as the possible regulatory mechanisms were investigated.

## 2 Materials and methods

### 2.1 Clinical samples

A total of 30 patients with CC were recruited in Maternal and Child Health Hospital of Hubei Province. CC tissues and paired adjacent normal tissues were obtained during the excision surgery and kept in a liquid nitrogen container immediately. All participants had not received radiotherapy, chemotherapy, or any other treatment before operation.


**Informed consent:** Informed consent has been obtained from all individuals included in this study.
**Ethical approval:** The research related to human use has been complied with all the relevant national regulations and institutional policies and in accordance with the tenets of the Helsinki Declaration, and has been approved by the Ethics Committee of Maternal and Child Health Hospital of Hubei Province.

### 2.2 Cell culture and transfection

Human normal immortalized cervical epithelial cell line (H8) was purchased from Institute of Preclinical Medicine, Peking Union Medical University (Beijing, China), and CC HeLa (CCL-2) and SIHA (HTB-35) cells were acquired from the American Type Culture Collection (ATCC, Rockville, MD, USA). The aforementioned cells were maintained in Dulbecco’s Modified Eagle Medium (DMEM; HyClone, Logan, UT, USA) supplemented with 10% fetal bovine serum (FBS; Gibco BRL, Gran Island, NY, USA), 100 U/mL penicillin, and 100 μg/mL streptomycin (Sigma-Aldrich, St. Louis, MO, USA) at 37°C with 5% CO_2_ and 95% air.

Small interference RNA (siRNA) targeting PVT1 (si-PVT1) and its negative control (si-NC), miR-503 inhibitor (anti-miR-503) and its negative control miR-NC inhibitor (anti-miR-NC), and miR-503 mimic (miR-503) and its negative control miR-NC mimic (miR-NC) were designed and synthesized by GenePharma Co., Ltd (Shanghai, China). For upregulation of PVT1 and ARL2, corresponding overexpression plasmids pcDNA-PVT1 (PVT1) and pcDNA-ARL2 (ARL2) were constructed by Hanbio Biotechnology Co., Ltd (Shanghai, China), with non-targeting plasmid (pcDNA) as negative control. The aforementioned oligonucleotides or plasmids were transfected into CC HeLa and SIHA cells using Lipofectamine 3000 (Life Technologies Corporation, Carlsbad, CA, USA) based on the user’s manual.

### 2.3 Quantitative reverse transcription polymerase chain reaction (qRT-PCR)

Total RNA from CC tissues and paired adjacent normal tissues, CC cells (HeLa and SIHA), and H8 cells was isolated using the RNA Isolation Kit (Sigma-Aldrich). As for complementary DNA (cDNA) synthesis, 1 μg RNA was used, with the help of High Capacity cDNA Reverse Transcription Kit (Applied Biosystems, Foster City, CA, USA) or TaqMan miRNA Reverse Transcription Kit (Applied Biosystems). For detecting PVT1 and ARL2 mRNA expression, SYBR Green Real-Time PCR Master Mix (Roche Diagnostics, Basel, Switzerland) was selected for qPCR. For miR-503 analysis, the all-in-one miRNA RT-qPCR Detection Kit (GeneCopoeia Inc., Rockville, MD, USA) was used. In addition, qPCR was operated on ABI PRISM 7500 real-time PCR System (Applied Biosystems). Relative expression of PVT1, miR-503, and ARL2 was evaluated with the 2^−ΔΔCt^ method. Glyceraldehyde-3-phosphate dehydrogenase (GAPDH) was used to normalize PVT1 and ARL2 expression, and U6 served as an endogenous control for miR-503. The sequences of primers involved in the qRT-PCR study are listed as follows: PVT1, forward 5′-GCCCCTTCTATGGGAATCACTA-3′ and reverse 5′-GGGGCAGAGATGAAATCGTAAT-3′; ARL2, forward 5′-GAAGCAGAAAGAGCGGGA-3′ and reverse 5′-CTGTGAAAATGCGGCTGGA-3′; GAPDH, forward 5′-GGAGCGAGATCCCTCCAAAAT-3′ and reverse 5′-GGCTGTTGTCATACTTCTCATGG-3′; miR-503, forward 5′-ACTGGCCTAAGTACACCCAGT-3′ and reverse 5′-GCTGCGAAGTGGAAACCATC-3′; and U6, forward 5′-CTCGCTTCGGCAGCACA-3′ and reverse 5′-AACGCTTCACGAATTTGCGT-3′.

### 2.4 3-(4,5)-Dimethylthiazole-2-y1)-2,5-biphenyl tetrazolium bromide (MTT) assay

For cell proliferation assessment, MTT assay was conducted. Briefly, after transfection HeLa and SIHA cells were seeded into 96-well plates and maintained in DMEM with 10% FBS for 24, 48, and 72 h. Then, 10 μL of MTT (5 mg/mL; Sigma-Aldrich) was added, dropwise, into each well. After 4 h absorbance of each well at 490 nm was determined on a Microplate Reader (Bio-Rad, Hercules, CA, USA).

### 2.5 Cell apoptosis assay

Apoptosis rate of transfected HeLa and SIHA cells was analyzed using Annexin V-fluorescein isothiocyanate (FITC) Apoptosis Detection kit (BD Biosciences, Franklin Lakes, NJ, USA) in compliance with the protocols supplied by the manufacturer. At 48 h post transfection, cells were collected and resuspended in 1× binding buffer, and then stained with 5 µL of Annexin V-FITC and 5 µL of propidium iodide (PI) for 15 min away from light. Afterward, cell apoptosis rate was monitored with a flow cytometer (Beckman Coulter, Fullerton, CA, USA).

### 2.6 Transwell migration and invasion assays

For evaluation of cell migration and invasion abilities, Transwell chamber (8 μm; BD Biosciences) was used. As for invasion detection, HeLa or SIHA cells (1 × 10^5^) were seeded into upper chamber pre-coated with Matrigel (BD Biosciences), with DMEM inside, whereas upper chamber without Matrigel was selected for migration analysis. Meanwhile, DMEM supplemented with 20% FBS was placed into the lower chamber. After 24 h of maintenance at 37°C, cells remaining on the supine surface of the insert were removed using sterile swab. The cells that went through the Transwell membrane were fixed, stained, and then counted under an optical microscope (Olympus, Tokyo, Japan).

### 2.7 Dual luciferase reporter assay

The miRNAs interacted with PVT1 and target genes of miR-503 were predicted by online software miRcode and Target Scan Human 7.2, respectively. The wide-type luciferase reporters (WT-PVT1 and WT-ARL2) were generated by cloning fragments of PVT1 and ARL2 3′-UTR harboring binding sites (5′-GCUGCUA-3′) with miR-503 into pGL3 luciferase promoter vectors (Promega, Madison, WI, USA) according to the manufacturer’s instructions. Likewise, mutant ones were established by inserting fragments containing the corresponding mutant-binding sites (5′-CGACGAU-3′). These reporters were severally cotransfected into CC HeLa and SIHA cells with pRL-TK Vector (Promega; an internal control) and miR-503 or miR-NC using Lipofectamine 3000 (Life Technologies). After 48 h, luciferase activity was measured using Dual-Luciferase Reporter detection System (Promega).

### 2.8 Western blot

CC tissues, adjacent normal tissues, H8 cells as well as CC HeLa and SIHA cells were lysed in radioimmunoprecipitation assay (RIPA) buffer (Thermo Fisher Scientific, Inc., Waltham, MA, USA) containing protease inhibitor (Thermo Fisher Scientific) for protein isolation. After concentration determination using bicinchoninic acid protein assay kit (Sigma-Aldrich), protein samples (20 μg) were loaded on fresh sodium dodecyl sulfate-polyacrylamide gel electrophoresis (SDS-PAGE, 10%), and then electro-transferred onto a polyvinylidene difluoride membrane (PVDF; Millipore, Billerica, MA, USA). After blockage with 5% defatted milk, the membrane was probed at 4°C overnight with primary antibody against ARL2 (ab183510, 1:1,000 dilution; Abcam, Cambridge, MA, USA) or GAPDH (ab8245, 1:3,000 dilution; Abcam). Subsequently, the protein blots were incubated with secondary antibody (ab205718, 1:5,000 dilution; Abcam) at indoor temperature for 2 h, and then visualized using Millipore ECL western blot detection system (Millipore). Density of protein blots was analyzed using Image J software (NIH, Bethesda, MD, USA) normalized to GAPDH.

### 2.9 *In vivo* experiment

To investigate the functional effect of PVT1 on CC tumor growth, *in vivo* experiment was performed. Short hairpin RNA (shRNA) targeting PVT1 (sh-PVT1) and its negative control (sh-NC) synthesized by GenePharma Co. Ltd were stably transfected into SIHA cells. Five-week-old female athymic BALB/c nude mice (*n* = 5; Shanghai Experimental Animal Center of the Chinese Academy of Sciences, Shanghai, China) were hypodermically injected with stably transfected SIHA cells (2 × 10^6^/0.2 mL PBS) in the right back of each individual. The tumor volume was monitored with a caliper and calculated (0.5 × length × width^2^) every 4 days. After 27 days all mice were killed, and the tumors were excised for weight and evaluation of expression levels of PVT1, miR-503, and ARL2.


**Ethical approval:** The research related to animal use has been complied with all the relevant national regulations and institutional policies for the care and use of animals and has been approved by the Animal Care and Use Committee of Maternal and Child Health Hospital of Hubei Province.

### 2.10 Statistical analysis

Data in this study from at least three independent experiments were processed with SPSS 21.0 statistical software (SPSS, Chicago, IL, USA). All data were exhibited as mean ± standard deviation. Difference was determined by Student’s *t*-test (for data between two groups) or one-way analysis of variance (for data among three groups). The statistically significant difference indicated *P* value <0.05. The correlation between lncRNA PVT1 expression and the clinicopathological features of CC patients (Table 1) was analyzed via the chi-square test (*χ* ^2^ test).

**Table 1:** Correlation analysis of lncRNA PVT1 expression with the clinicopathological features of CC patients

Parameters	Number of cases	Lnc-PVT1		*P*-value
		Low	High	
**Age (years)**				
<60	8	2	6	0.412
≥60	22	8	16	
**Menopause**				
Yes	6	3	3	0.624
No	24	8	16	
**Tumor size (cm)**				
<3	13	6	7	0.272
≥3	17	6	11	
**Differentiation**				
Well/moderate	19	5	14	0.337
Poor	11	5	6	
**TNM stage**				
I + II	12	4	8	0.2
III	18	7	11	

TNM: Tumor node metastasis.

## 3 Results

### 3.1 PVT1 was upregulated in CC tissues and cell lines

**Figure 1:**
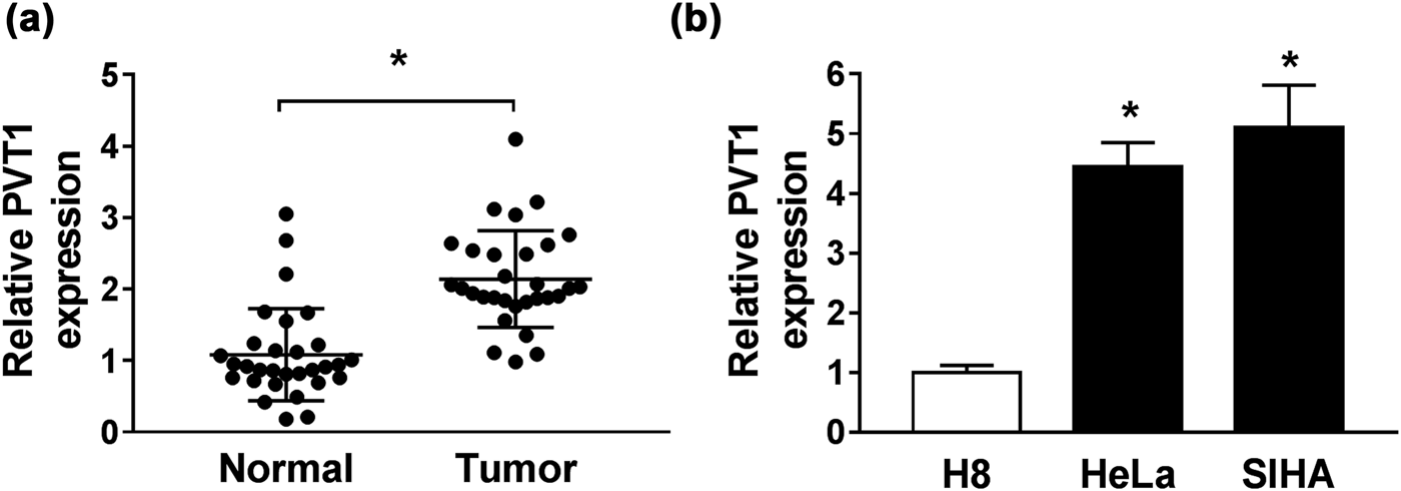
PVT1 was upregulated in CC tissues and cell lines. (a and b) The expression of PVT1 in CC tissues and paired adjacent normal tissues (a), as well as in CC cells (HeLa and SIHA) and H8 cells (b) examined by qRT-PCR assay. *P < 0.05. All experiments were repeated thrice, independently.

For determination of the role of PVT1 in CC progression, qRT-PCR assay was implemented to detect the enrichment of PVT1 in CC tissues and paired adjacent normal tissues, as well as in CC cells (HeLa and SIHA) and H8 cells. The result revealed that relative PVT1 expression was higher in CC tissues and cells in contrast to the corresponding controls (Figure 1a and b).

### 3.2 Silencing of PVT1 inhibited CC progression

**Figure 2:**
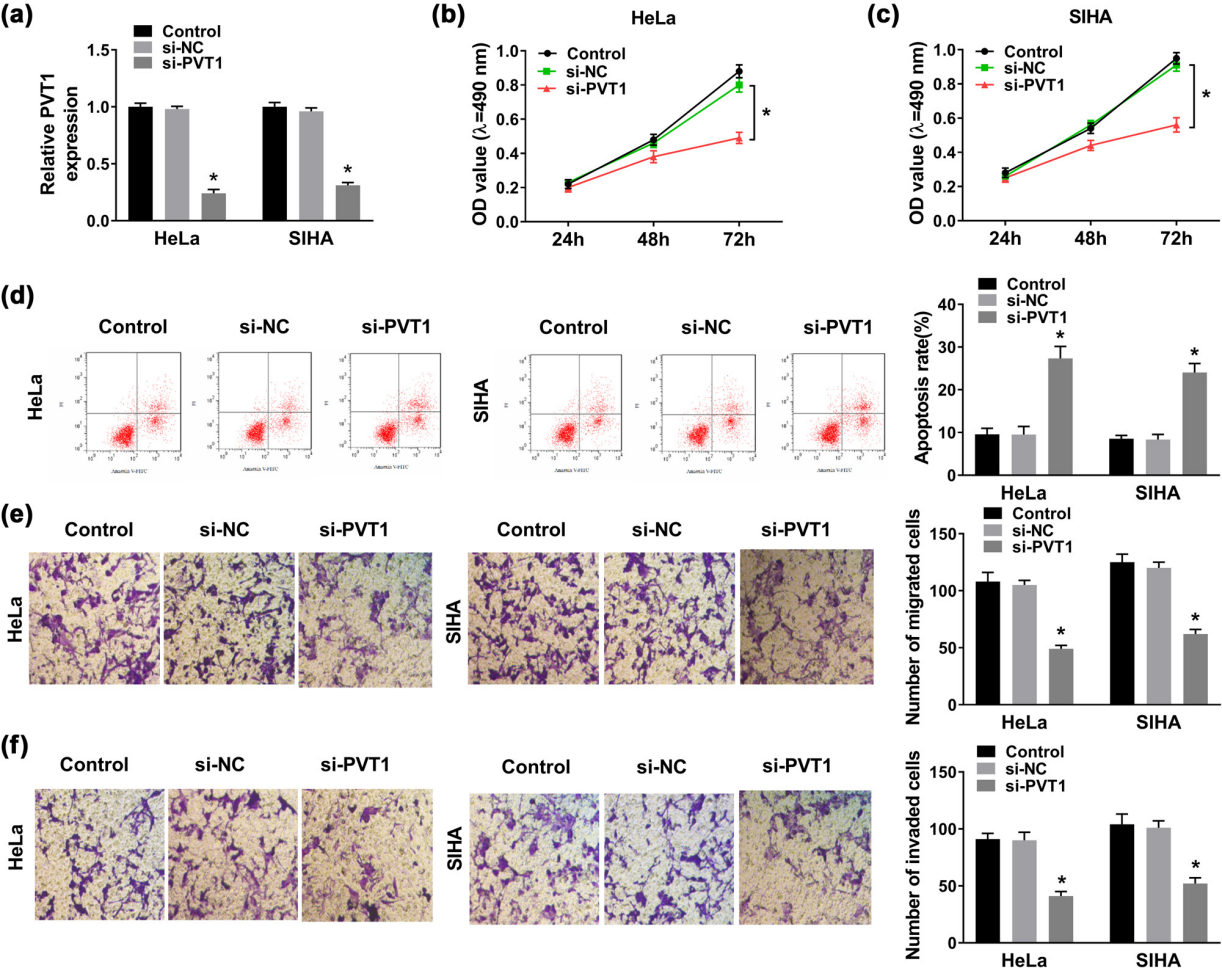
Silencing of PVT1 inhibited CC progression. CC HeLa and SIHA cells were transfected with control (blank), si-NC, or si-PVT1. (a) PVT1 expression in transfected HeLa and SIHA cells evaluated via qRT-PCR assay at 48 h post transfection. (b and c) Cell viability in HeLa and SIHA cells analyzed by MTT assay at 24, 48, and 72 h post transfection. (d) Cell apoptosis rate of transfected HeLa and SIHA cells evaluated by flow cytometry. (e and f) Cell migration and invasion examined by Transwell assay. *P < 0.05. All experiments were repeated thrice, independently.

To figure out whether PVT1 was involved in cellular behaviors of CC cells, we first transfected si-PVT1 or si-NC into CC HeLa and SIHA cells. Then qRT-PCR was conducted to validate the transfection efficiency, and the data manifested that there was a striking decrease in PVT1 expression in CC HeLa and SIHA cells of the si-PVT1 group, compared with that in the si-NC group (Figure 2a). Silencing of PVT1 resulted in an obvious reduction in cell viability of HeLa and SIHA cells transfected with si-PVT1 relative to cells transfected with si-NC (Figure 2b and c), which was proved by MTT assay. As for cell apoptosis, the results of flow cytometry suggested that PVT1 knockdown contributed to cell apoptosis of CC cells (Figure 2d). Obviously, silencing of PVT1 also retarded migration and invasion abilities of HeLa and SIHA cells, when compared to the si-NC group (Figure 2e and f). The aforementioned findings implied that PVT1 knockdown hampered CC progression *in vitro*.

### 3.3 PVT1 directly interacted with miR-503

**Figure 3:**
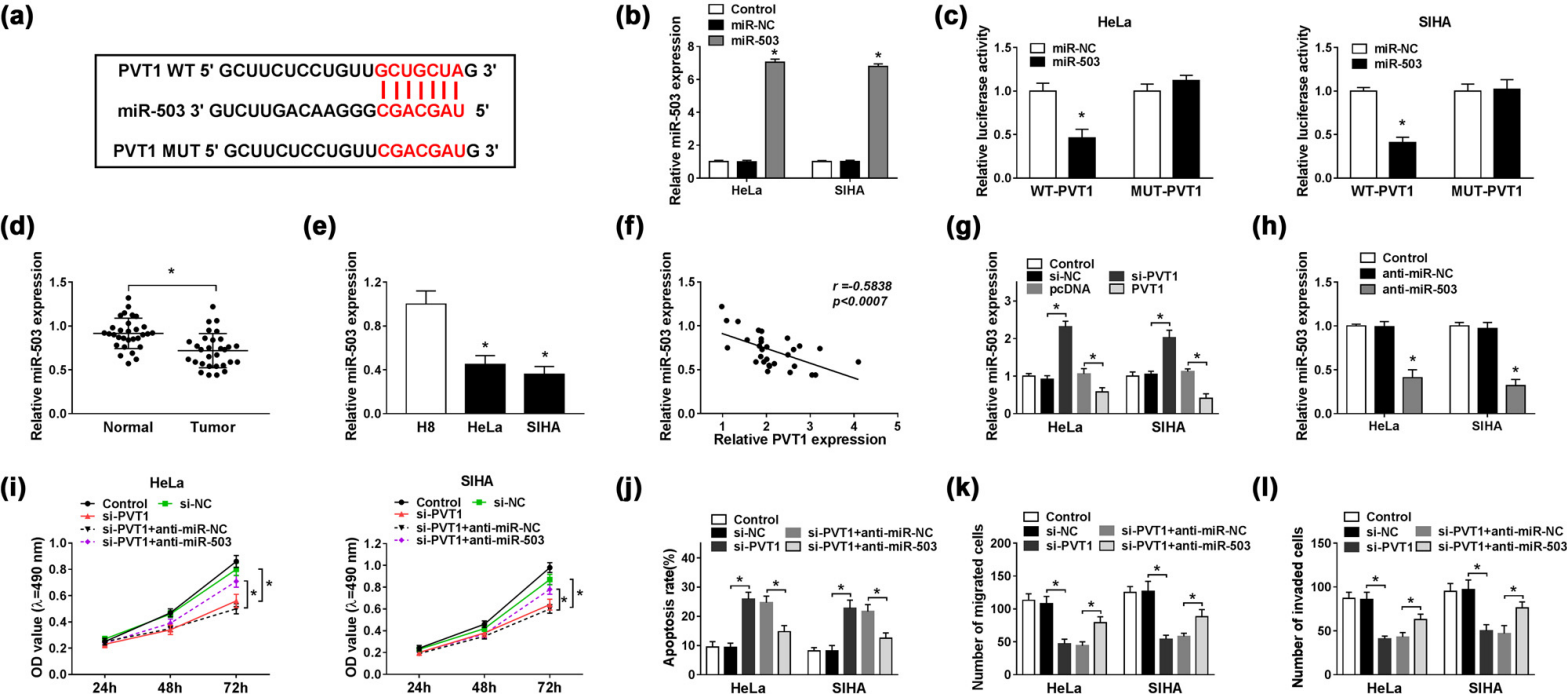
PVT1 directly interacted with miR-503. (a) The potential binding sites between PVT1 and miR-503 predicted by miRcode. (b) MiR-503 expression in HeLa and SIHA cells transfected with control (blank), miR-NC, or miR-503 detected by qRT-PCR assay. (c) Dual luciferase reporter assay for the luciferase activity of WT-PVT1 and MUT-PVT1 in HeLa and SIHA cells transfected with miR-503 or miR-NC at 48 h post transfection. (d and e) MiR-503 level in CC tissues and paired adjacent normal tissues (d), as well as in CC cells (HeLa and SIHA) and H8 cells (e) determined by qRT-PCR assay. (f) Correlation analysis for levels of miR-503 and PVT1 in CC tissues. (g) MiR-503 enrichment in HeLa and SIHA cells transfected with control (blank), si-NC, si-PVT1, pcDNA, or PVT1 detected by qRT-PCR assay. (h) Relative miR-503 expression in HeLa and SIHA cells treated with control (blank), anti-miR-NC, or anti-miR-503 evaluated by qRT-PCR assay. (i–l) HeLa and SIHA cells were transfected with control (blank), si-PVT1, si-PVT1 + anti-miR-NC, or si-PVT1 + anti-miR-503 for 48 h. (i) Cell viability of treated CC cells examined via MTT assay. (j) Cell apoptosis of transfected CC cells monitored by flow cytometry. (k and l) Cell migration and invasion capacities evaluated by Transwell assay. *P < 0.05. All experiments were repeated thrice, independently.

**Figure A1:**
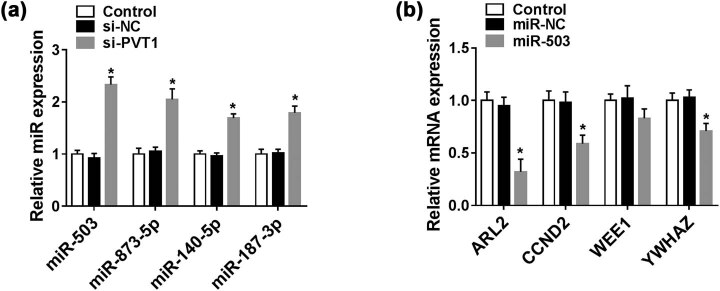
Selection of target of PVT1 and miR-503. (A) QRT-PCR assay for the expression levels of miR-503, miR-873-5p, miR-140-5p, and miR-187-3p in SIHA cells transfected with control (blank), si-NC, or si-PVT1. (B) QRT-PCR assay for the expression levels of ARL2, CCND2, WEE1, and YWHAZ in SIHA cells transfected with control (blank), miR-NC, or miR-503. *P < 0.05. All experiments were repeated thrice, independently.

Generally speaking, lncRNAs exert their regulatory roles by serving as sponges for miRNAs. In the current study, we’ve searched for downstream miRNAs of PVT1 using miRcode software, and found that PVT1 could bind to miR-503, miR-873-5p, miR-140-5p, or miR-187-3p. In addition, the most significant upregulation among these four miRNAs was discovered in miR-503 expression in SIHA cells transfected with si-PVT1 (Figure A1a). Therefore, miR-503 was selected for the following assays, and the binding sites between PVT1 and miR-503 are shown in Figure 3a. Introduction of miR-503 efficiently increased its expression in HeLa and SIHA cells, when compared to miR-NC (Figure 3b). Subsequent dual luciferase reporter assay further confirmed the targeted relationship between PVT1 and miR-503, reflected by the reduced luciferase activity of PVT1 WT in both HeLa and SIHA cells, whereas no significant change was observed in the luciferase activity of PVT1 MUT (Figure 3c). Next, we analyzed the miR-503 level in CC tissues and cell lines and found that miR-503 expression was apparently reduced in CC (Figure 3d and e). Pearson analysis manifested that miR-503 expression in CC tissues was negatively correlated with the PVT1 level (*r* = −0.5838, *P* < 0.0007; Figure 3f). Then, we explored the effect of PVT1 on miR-503 expression and discovered that silencing of PVT1 upregulated miR-503 expression in HeLa and SIHA cells; in contrast, introduction of PVT1 drastically reduced the miR-503 level (Figure 3g). By transient transfection with anti-miR-503, the miR-503 expression was successfully downregulated in CC cells, shown by qRT-PCR assay (Figure 3h). As exhibited in Figure 3i, transfection of si-PVT1 notably hampered cell viability of transfected HeLa and SIHA cells, but simultaneous introduction of anti-miR-503 almost abolished the inhibitory impact. Flow cytometry assay indicated that PVT1 knockdown-induced promotion of cell apoptosis was weakened by downregulation of miR-503 (Figure 3j), and partially reversed effects were also observed in cell migration and invasion abilities in HeLa and SIHA cells cotransfected with si-PVT1 and anti-miR-503 (Figure 3k and l). In short, PVT1 knockdown might hamper CC progression by increasing miR-503 expression *in vitro*.

### 3.4 ARL2 was a direct target of miR-503, and PVT1 upregulated ARL2 by sponging miR-503

**Figure 4:**
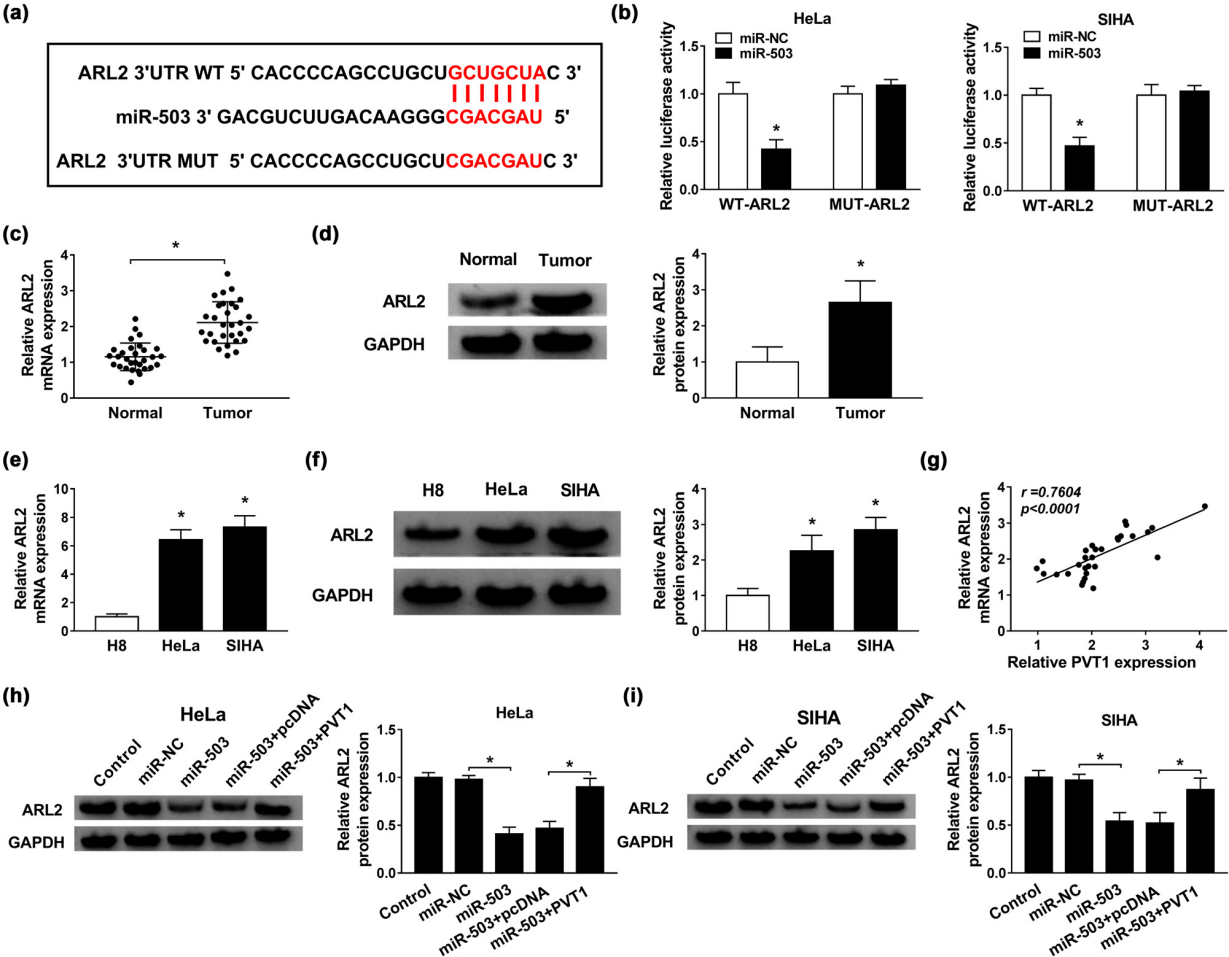
ARL2 was a direct target of miR-503, and PVT1 upregulated ARL2 by sponging miR-503. (a) The binding region between miR-503 and ARL2 predicted by TargetScanHuman 7.2. (b) Dual luciferase reporter assay for the luciferase activities of WT-ARL2 and MUT-ARL2 in HeLa and SIHA cells transfected with miR-503 or miR-NC. (c–f) The mRNA and protein expression levels of ARL2 in CC tissues and cell lines, as well as in the corresponding controls. (g) Correlation analysis for enrichment of ARL2 and PVT1 in CC tissues. (h and i) Western blot assay for protein level of ARL2 in HeLa and SIHA cells transfected with control (blank), miR-NC, miR-503, miR-503 + pcDNA, or miR-503 + PVT1. *P < 0.05. All experiments were repeated thrice, independently.

In addition, we made efforts to seek for the downstream genes of miR-503 with the aid of TargetScanHuman 7.2. We’ve found a binding region between miR-503 and ARL2, CCND2, WEE1, or YWHAZ. SIHA cells transfected with miR-503 presented the greatest decrease in ARL2 level compared with the other predicted genes (Figure A1b). Thus, we chose ARL2 as a target of miR-503 for later investigation. The binding position between miR-503 and ARL2 3′-UTR is exhibited in Figure 4a. Then, dual luciferase reporter assay was used to validate the interaction between miR-503 and ARL2. MiR-503 greatly reduced the luciferase activity of WT-ARL2 in both HeLa and SIHA cells, but not the MUT-ARL2 (Figure 4b). Subsequently, qRT-PCR and western blot assays were performed to measure the mRNA and protein expression levels of ARL2 in CC tissues and cell lines, and the results implied that ARL2 exhibited high expression in CC tissues and cell lines in contrast to the corresponding controls (Figure 4c–f). As expected, the ARL2 level in CC tissues was positively correlated with the PVT1 level (*r* = 0.7604, *P* < 0.0001; Figure 4g). Moreover, western blot assay further proved that overexpression of miR-503 triggered an obvious reduction of ARL2 enrichment, which was largely recovered by gain of PVT1 in transfected HeLa and SIHA cells (Figure 4h and i). In conclusion, PVT1 mediated ARL2 expression by sponging miR-503 in CC.

### 3.5 Overexpression of ARL2 almost abrogated PVT1 deficiency-mediated anti-proliferation, pro-apoptosis, and anti-metastasis effects on CC cells

**Figure 5:**
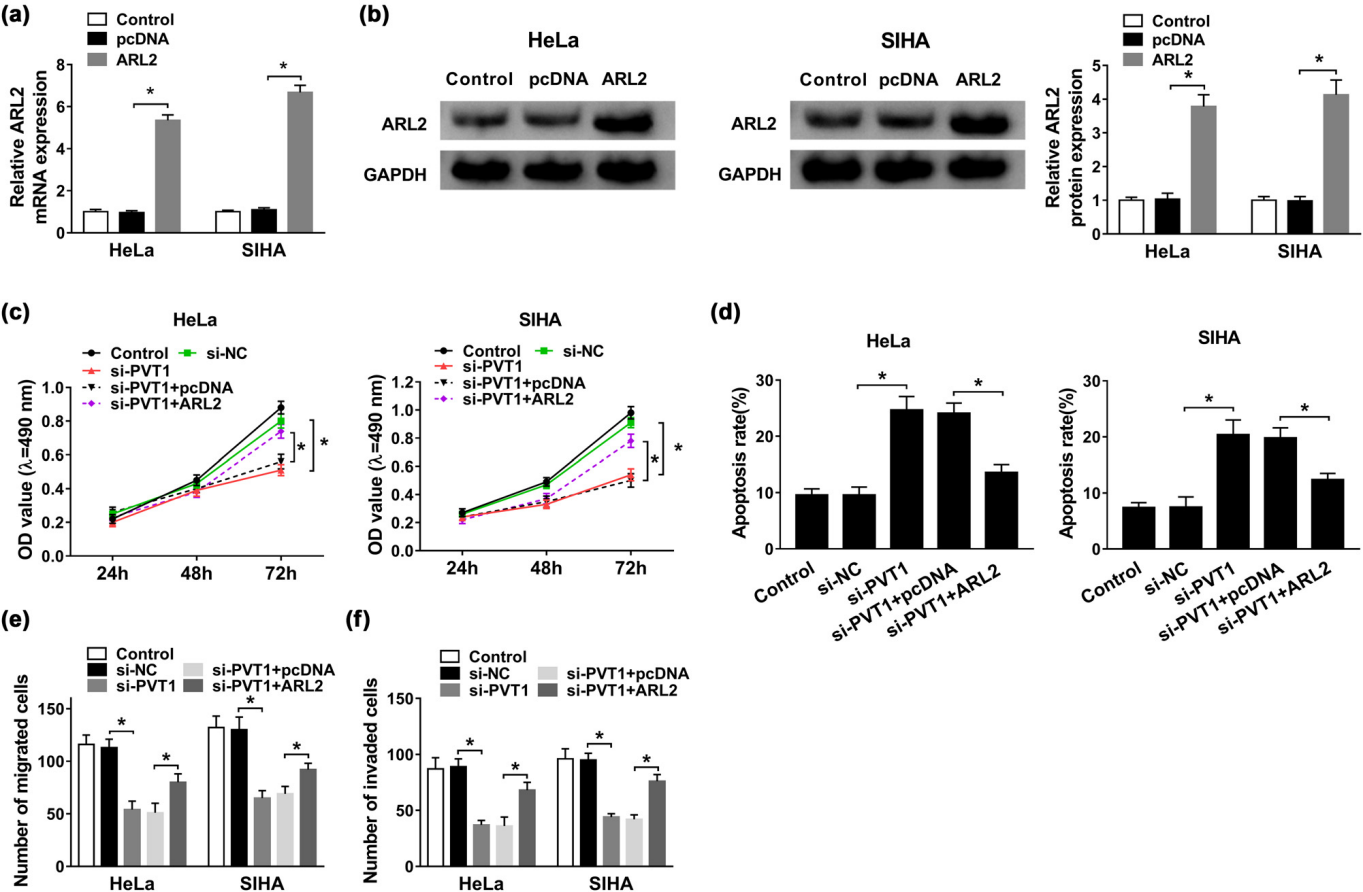
Overexpression of ARL2 almost abrogated PVT1 deficiency-mediated anti-proliferation, pro-apoptosis, and anti-metastasis effects on CC cells. (a and b) QRT-PCR and western blot assays for the mRNA (a) and protein (b) expression of ARL2 in HeLa and SIHA cells transfected with control (blank), pcDNA, or ARL2, respectively. (c–f) HeLa and SIHA cells were transfected with control (blank), si-NC, si-PVT1, si-PVT1 + pcDNA, or si-PVT1 + ARL2. (c) MTT assay for examination of the cell viability of HeLa and SIHA cells. (d) Cell apoptosis evaluation for transfected HeLa and SIHA cells by flow cytometry. (e and f) Transwell assay for migration and invasion of treated HeLa and SIHA cells. *P < 0.05. All experiments were repeated thrice, independently.

As depicted in Figure 5a, ARL2 mRNA expression was evidently higher in HeLa and SIHA cells transfected with ARL2 than that in the pcDNA group. Furthermore, upregulation of ARL2 protein level was also detected as shown by western blot assay (Figure 5b). Then, MTT assay revealed that upregulation of ARL2 largely relieved the decreased cell viability in both HeLa and SIHA cells caused by si-PVT1 (Figure 5c). Besides, upregulation of ARL2 attenuated the si-PVT1-induced apoptosis promotion, which was evidenced by flow cytometry (Figure 5d). In addition, the overexpression of ARL2 alleviated the repressive effects of PVT1 knockdown on cell migration and invasion of CC cells (Figure 5e and f). In a word, the anti-proliferation, pro-apoptosis, and anti-metastasis effects on CC cells induced by PVT1 deficiency were all weakened by overexpression of ARL2.

### 3.6 PVT1 inhibition suppressed CC tumor growth *in vivo*

**Figure 6:**
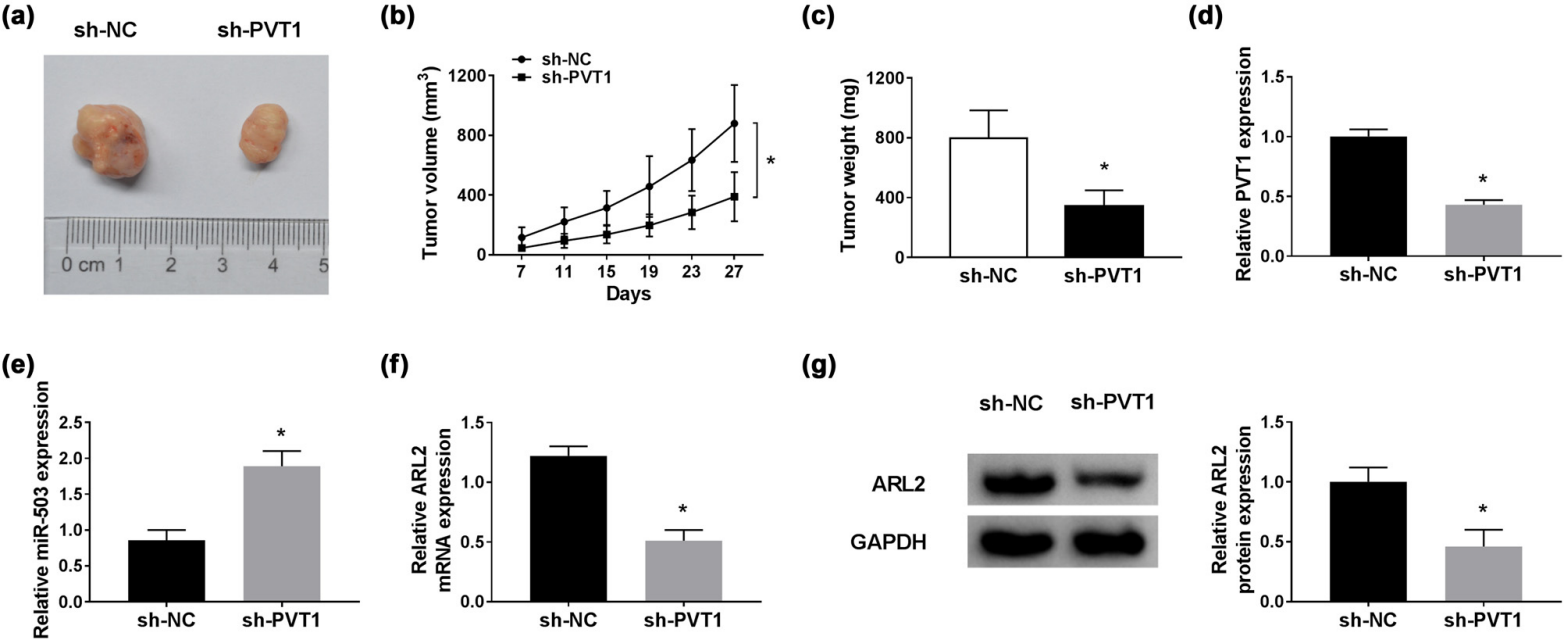
PVT1 inhibition suppressed CC tumor growth *in vivo*. (a) Images of excised tumor tissues. (b) The tumor volume was calculated using the formula (0.5 × length × width^2^) once per 4 days. (c) The weight of excised tumor tissues at 27 days post injection. (d and e) QRT-PCR assay for PVT1 and miR-503 expression in excised tumor tissues. (f and g) QRT-PCR and western blot assays for ARL2 level in excised tumor tissues. *P < 0.05. All experiments were repeated thrice, independently.

In addition, the effect of PVT1 on CC tumor growth was determined *in vivo*. As shown in Figure 6a–c, PVT1 deficiency curbed the tumor growth (Figure 6a and b) and reduced tumor weight (Figure 6c), in contrast to mice injected with CC SIHA cells stably transfected with sh-NC. Then the expression of PVT1, miR-503, and ARL2 was examined in the resected tumor tissues. The data indicated that PVT1 expression was reduced (Figure 6d), whereas miR-503 expression was increased (Figure 6e) in the sh-PVT1 group relative to the sh-NC group. Furthermore, ARL2 was downregulated in the sh-PVT1 group at both mRNA and protein level, when compared to the sh-NC group (Figure 6f and g). Taken together, these results indicated that PVT1 depletion suppressed CC tumor growth *in vivo*.

## 4 Discussion

**Figure 7:**
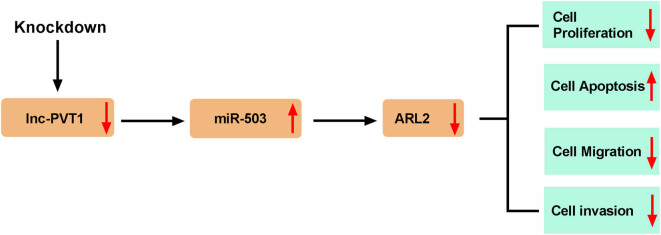
Schematic diagram of PVT1 regulating the proliferation, apoptosis, migration, and invasion of CC cells. Knockdown of PVT1 inhibited proliferation, migration, and invasion, and facilitated apoptosis of CC cells by regulating the miR-503/ARL2 axis.

As an intractable malignancy, CC presents a great threat to women’s health. This issue is especially common among young women of low- and middle-income countries [25]. In this study, we observed an obvious upregulation of PVT1 expression in CC tissues and cell lines, and silencing of PVT1 restricted cell proliferation, migration and invasion *in vitro*, as well as inhibited tumor growth *in vivo*. We further explored the targeted relationship among PVT1, miR-503, and ARL2 and draw the conclusion that PVT1 directly targeted miR-503 and that ARL2 was the direct target of miR-503. Experimental data suggested that PVT1 could induce ARL2 expression by sponging miR-503, and the involvement of PVT1/miR-503/ARL2 axis in CC progression was shown for the very first time (Figure 7).

PVT1 was shown to play an oncogenic role as an inducer of progression of certain human cancers. It was reported that depletion of PVT1 expression resulted in the reduction of migration and invasion capacities of small cell lung cancer (SCLC) cells *in vitro* [26]. Zhao and his partners declared that PVT1 was a sponge of miR-448 to upregulate SERBP1, thereby facilitating proliferation and migration of pancreatic cancer cells [12]. In esophageal cancer, highly expressed PVT1 significantly promoted invasion of TE-1 and Eca-109 cells by accelerating the epithelial-to-mesenchymal transition (EMT) process [13]. *In vitro* assay conducted by Yang et al. implied that PVT1 was obviously upregulated in NSCLC tumor tissues, and lack of PVT1 conspicuously suppressed cell proliferation, migration, and invasion of NSCLC cells [11]. Likewise, upregulation of PVT1 was also detected in CC tissues, especially in tumors at higher FIGO stage [27]. In our study, a remarkable increase in PVT1 expression was detected in CC tissues and CC cells (HeLa and SIHA), as shown previously [27,28]. *In vivo* assay confirmed that PVT1 knockdown could repress CC tumor growth.

LncRNA PVT1 was corroborated to be a potential therapeutic target for CC, and PVT1 proved its tumor-promoting role by negatively modulating miR-424 [28]. The oncogenic role of PVT1 was also identified by its knockdown-mediated significant decrease in proliferation, migration, and invasion, as well as significant increase in apoptosis and cisplatin cytotoxicity in CC SIHA cells [29]. To gather evidence, we performed MTT, flow cytometry, and transwell assays and observed analogous results in both HeLa and SIHA cells transfected with si-PVT1.

Subsequently, we tried to investigate the exact regulatory mechanism by which PVT1 participates in CC progression. Online software miRcode was used to seek for the targeted miRNAs of PVT1, and identified miR-503 as a candidate, which was further validated by dual luciferase reporter assay. MiR-503 acted as a tumor suppressor in endometrioid endometrial cancer (EEC), and its relative level was positive with the survival time of patients with EEC [30]. In addition, miR-503 was downregulated in prostate cancer tissues while acting as an inhibitor of proliferation and metastasis of prostate cancer cells by targeting RNF31 [31]. Besides, it was reported that miR-503 suppressed cell proliferation of breast cancer MCF-7 cells by directly targeting oncogene ZNF217, acting as a tumor suppressor miRNA [32]. Our experimental data revealed that miR-503 was distinctly downregulated in CC tissues and cells, in concordance with the outcome of Yin et al. [33]. In addition, the miR-503 level was passively regulated by PVT1 and was negatively correlated with PVT1 expression in CC tissues. Moreover, miR-503 inhibition almost rescued the si-PVT1-mediated repressed impact on cell proliferation, migration, and invasion of CC HeLa and SIHA cells.

Then, ARL2 was recognized as a target of miR-503 by bioinformatics analysis using TargetScanHuman 7.2 what was later confirmed by dual luciferase reporter assay. ARL2 was reported to cooperate with miR-214 in regulation of the carcinogenesis of colon cancer [23] and CC [24]. Sun et al. showed that ARL2 was a novel target of miR-497-5p, and ARL2 knockdown contributed to cell apoptosis and impeded cell proliferation of osteosarcoma MG-63 and U2OS cells [22]. Furthermore, interference of ARL2 had inhibitory effects on migration and invasion of CC cells, suggesting the oncogenic role of ARL2 [34]. From our results, both the mRNA and protein expression levels of ARL2 in CC tissues and cell lines were prominently upregulated, which is consistent with the previously published papers [24,34]. Moreover, ARL2 expression was positively correlated with PVT1 expression in CC tissues. Functionally, overexpressed ARL2 evidently ameliorated the silencing of PVT1-mediated anti-proliferation and anti-metastasis effects on CC cells. Reportedly, ARL2 could affect breast tumor growth and aggressiveness via PP2A-mediated pathway [35]. Whether ARL2 influences CC progression by regulating PP2A content and activity remains to be discovered.

Taken together, our data manifested that silencing of lncRNA PVT1 repressed proliferation and metastasis of CC cells *in vitro*, as well as inhibited tumorigenesis *in vivo*. In addition, PVT1 plays a role in CC progression by regulating the miR-503/ARL2 axis, at least in part. Our investigation might provide therapeutic targets for the treatment of CC patients.
